# Environmental performance of a multi-energy liquid air energy storage (LAES) system in cogeneration asset – A life cycle assessment-based comparison with lithium ion (Li-ion) battery

**DOI:** 10.1016/j.heliyon.2024.e39193

**Published:** 2024-10-10

**Authors:** Alessio Tafone, Emiliano Borri, Luisa F. Cabeza, Alessandro Romagnoli

**Affiliations:** a TUMCREATE, 1 CREATE Way, #10-02 CREATE Tower, 138602, Singapore; b GREiA Research Group, Universitat de Lleida, Pere de Cabrera s/n, 25001, Lleida, Spain; c School of Mechanical and Aerospace Engineering, Nanyang Technological University, Singapore

**Keywords:** Liquid air energy storage (LAES), Life cycle assessment (LCA), Electrical energy storage, Carnot battery, Li-ion battery, Thermal energy storage (TES), Cogeneration, District cooling

## Abstract

Increase in energy demand is shaping both developed and developing countries globally. As a result, the endeavour to reduce carbon emissions also encompasses electrical energy storage systems to ensure environmentally friendly power production and distribution. Currently, the scientific community is actively exploring and developing new storage technologies for this purpose. The focus of this work is to compare the eco-friendliness of a relatively novel technology such as liquid air energy storage (LAES) with an established storage solution such as Li-Ion battery (Li-ion). The comparison is carried out through Life Cycle Assessment (LCA) methodology which aims to assess the environmental impacts from each life stage, according to different impact categories. In particular, the study refers to the unitary storage and the delivery of electricity as well as cooling energy, considering all the inefficiencies and limits of each technology. The results show that in the full electric case study Li-ion battery environmentally outperform LAES due to (1) the higher round trip efficiency and (2) the significantly high environmental impact of the diathermic oil utilized by LAES, accounting for 92 % of the manufacture and disposal phase. Conversely, the cogeneration case study highlights that the “flexibility” and “dualism” of LAES, capable to efficiently deliver both electricity and cooling, offset the impact related to the higher electricity consumption during the use phase. Notably in energy mix frameworks with high share of primary energy source from fossil fuels, cogenerative LAES demonstrates superior environmental performance compared to Li-ion battery (i.e. 1302 kg_CO2eq_/MWh_e_ vs 1140 kg_CO2eq_/MWh_e_ for Singapore energy mix), attributable to its reduced electricity consumption.

## 1 Introduction

Nowadays, policy makers are widely fostering a global shift towards low-carbon energy resources: the need to reduce CO_2_ emissions and the increase in energy security has become a primary target. One of the available solutions comes from renewable energy sources (RES) [1] even though, their nondeterministic nature (especially wind and solar which are expected to dominate the renewable electricity-mix in the future) will lead grid operators to constantly pursue the instant matching of demand and supply in order to guarantee the grid stability [2]. Electrical energy storage (EES) systems play a pivotal role in addressing the complex challenge linked to significantly augment the share of renewable sources into the future decarbonized power grid. Beyond their capability to mitigate grid instability, EES systems offer a distinctive ability to decouple electricity demand and supply, affording the strategic potential for adept peak-shaving operations during hours of heightened demand [3].

Currently, various technologies are available to store electric energy based on different storage mechanisms such as chemical or mechanical processes. The most widely known are pumped hydro storage, electro-chemical energy storage (e.g. Li-ion battery, lead acid battery, etc.), flywheels, and super capacitors. Energy storage systems that operate for hours at power ratings from Megawatt to Gigawatt play a crucial role in effectively integrating intermittent RES with limited regulation capability [4]. Within the category of grid-scale EESs, thermo-mechanical energy storage systems, commonly referred to as Carnot batteries in literature, currently present the lowest technology readiness level (TRL). Nonetheless, they are progressively gathering momentum in academic/industry investigations and discussions.

**Fig. 1:**
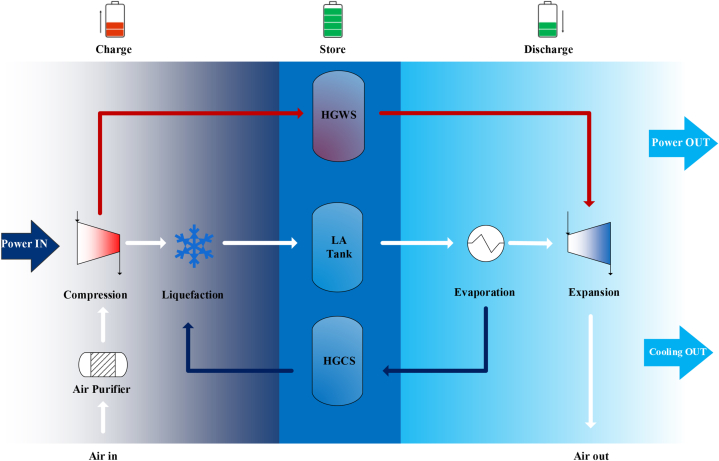
Liquid air energy storage concept. Adapted from Ref. [12].

Among Carnot batteries technologies such as compressed air energy storage (CAES) [5], Rankine or Brayton heat engines [6] and pumped thermal energy storage (PTES) [7], the liquid air energy storage (LAES) technology is nowadays gaining significant momentum in literature [8]. An important benefit of LAES technology is that it uses mostly mature, easy-to-source off-the-shelf components (i.e. turbo-machinery, pumps, and shell and tube and plate-fin heat exchangers) and does not depend on critical minerals as many batteries. Suitable for mid-to-large scale applications (10–150 MW_e_/80–7200 MWh) [5], LAES generally outperforms other Carnot battery technologies in terms of energy density (40–100 kWh/m^3^) and capital cost (900–6000 $/kW_e_) [5]. One of its key advantages is the ability to be deployed without geographical limitations, while also being environmentally safe [9]. The operation of the LAES system includes the typical three distinct phases, common to most of the EES systems (see Fig. 1): charging, storing, and discharging. The charge phase involves the liquefaction of ambient air through a cryogenic thermodynamic process (i.e. Linde and Kapitza, etc. [10]). The liquefied air is then stored at a specific pressure (approximately 1–10 bar [11]) in well-insulated vessels. During the discharge phase, a power recovery thermodynamic cycle is employed, utilizing the thermal energy from ambient air or potentially available waste heat, thus feeding the stored electrical energy back into the grid.

A real application of the LAES system was demonstrated in 2011 by Highview Power which developed and operated the first pilot plant (350 kW/2.5 MWh) [13], currently installed at the University of Birmingham (UK), and, subsequently in 2018 in collaboration with Viridor, the first grid scale demonstrator plant (5 MW/15 MWh) [14], capable to achieve a round trip efficiency up to 60 % due to the correct design and implementation of the cold thermal energy storage. In order to further scale-up the LAES capacity, Highview Power is currently aiming to develop the CRYObattery system, a LAES capable to deliver discharge power higher than 50 MW_e_. Cryo-battery projects were currently deployed in the UK and US [9] and may represent the first grid-connected LAES system in the world. Furthermore, within the umbrella of the partnership agreement between Highview Power and Sumitomo Heavy Industries (SHI) [15], two 50 MW LAES configurations (4 h and 8 h discharge periods) are planned to be deployed in China [16], paving the way for the commercialization of the plant also in Asia.

At R&D stage, the main focus areas are mostly directed toward the hybridization concept of LAES and the development of an efficient and cost-effective cold thermal energy storage solution, also known as high grade cold storage (HGSC). In terms of LAES hybridization, different configurations were proposed in literature. Antonelli et al. [17] analysed various hybrid configurations coupling LAES to ORC, and Brayton cycles, with or without additional combusted fossil fuels. Among these configurations, the cold Brayton cycle outperformed the other configurations, achieving a significant round trip efficiency of up to 90 %. A thermo-economic analysis for an energy storage system that combined a compressed air energy storage (CAES) with LAES components was carried out by Pimm et al. [18]. The study revealed that the proposed system proves to be more cost-effective than the individual stand-alone systems, namely the CAES and LAES, given specific conditions such as a storage duration exceeding 36 h. Beside the state-of-the-art configuration implemented by Highview power for the cold thermal energy storage [19], namely a sensible heat packed bed (pebbles), novel solutions were proposed in literature based on alternative materials and configurations. Ryu et al. [20] proposed a novel HGCS configuration based on the combination of sensible heat and latent heat materials. According to the authors, the cascaded configuration is capable to double the LAES round trip efficiency of the baseline case. Chen et al. [21] analysed the implementation of a single PCM layer as storage medium for HGCS system, obtaining a round trip efficiency as high as 54.2 %. In a similar fashion, Tafone et al. investigates the utilization of PCM both as a single medium [22] and as a PCM cascaded solution [23] concluding that the latter configuration might further enhance the round trip efficiency of the system by 6 % compared to the single PCM configuration.

Another notable area of techno-economic opportunity for LAES directly stems from its thermo-mechanical nature allowing the system to integrate any waste heat/cold source available as well as provide thermal energy for a potential district cooling/heating grid [24]. Indeed, the capability of LAES to store and deliver upon request both heat, cooling and power, independently from each other, was already positively evaluated in literature. Mazzoni et al. [25] demonstrated the economic viability of LAES as multi-energy system compared to batteries for a micro-grid application, with positive net present value reached after 7–11 years of operation. By exploiting the multi-energy system capability of LAES and leveraging a more flexible district operation, Vecchi et al. [26] showed that an increased LAES round trip efficiency can be achieved (from 47 % to 72.8 %) with a reduction of the operational costs up to 12 %. In a similar fashion, Esmaeilion et al. [27] investigated an extend concept of multi-energy LAES including a combined cooling, heating and power (CCCP) system and a desalination unit. From an economic perspective, the polygenerative LAES allows to achieve notable payback period and internal rate of return of 3.4 years and 32 %, respectively. Cui et al. [28] analysed the techno-economic feasibility of a multi-energy LAES for four case studies in China showing that the energy storage system has enhanced economic performance when operated in multi-generation mode with a pay-back period decrease up to 42 % compared to the full electric mode. Considering different opportunities for RES integration in food warehouses, Fikiin et al. [29] proposed a novel LAES based technology (CryoHub) to synergistically providing both electricity and cooling. The same research group [30] investigated a trigenerative LAES directly coupled with a food factory. The tight integration between the LAES and the user needs allows the multi-energy LAES to outperform the full electric LAES by increasing the global efficiency by 20 %. Exploiting the above mentioned thermo-mechanical nature of LAES, Wang et al. [31] designed a multi-functional LAES capable to deliver electricity, heating, and oxygen. Despite the lower round trip efficiency, the plant showed an improved economic performance compared to the stand-alone LAES with a shorter pay-back period due to the economic valorization of other commodities, such as heating and oxygen. Aiming at optimizing the multi-energy dispatch of electricity, heating and cooling, Yan et al. [32] adopted the information gap theory methodology. The novel approach allows to achieve operational cost savings of approximately 7 %.

As extensively described in this section, although all the literature studies mentioned above highlighted the paramount importance of the LAES capability to deliver a wider portfolio of additional energy service to the context where it operates, none of the works presented delved into an exhaustive environmental analysis, clearly identifying how the multi-energy potential of LAES system impact the environmental performance of the plant. Indeed, similar to any system, LAES, or more in general any EESs, necessitates a specific energy input for different life cycle stages: i) component manufacturing; ii) infrastructure and facility construction; iii) ongoing operational maintenance, and iv) eventual disassembly and disposal procedures carried out during the decommissioning phase. Therefore, in order to maintain a low environmental impact profile, it is of paramount importance to analyse at a life cycle level the energy consumption and emissions generated by the LAES [33]. In literature, few studies are systematically assessing the environmental impact of EESs [34,35]. Focusing on compressed air energy storage, Bouman et al. [36] assessed the environmental impact of a CAES integrated to an offshore wind farm considering both a diabatic and an adiabatic configuration. Results showed that the diabatic configuration has the highest environmental impacts due to the combustion of natural gas whereas the adiabatic configuration was more environmentally friendly. Denholm and Kulcinski [37] employed LCA methodology to evaluate the environmental impact of different energy storage systems, among which PHS, CAES, and electrochemical batteries (vanadium and sodium polysulphide electrolytes). Results showed that, amongst the technologies investigated, PHS and CAES are the most environmentally friendly options when paired with renewable energy sources and fossil fuels, respectively. Kapila et al. [38] investigated the efficiency (net energy ratio) and the emissions of diabatic and adiabatic CAES and PHS. In terms of greenhouse emissions, the highest environmental impact was due to the use phase, with the PHS technology presenting lower global emissions. Overall, research on the environmental performance of Carnot batteries, particularly in the context of liquid air energy storage, is still lacking [39]. In this context, the present work aims to overcome these research gaps in the literature and goes a step further, introducing the following novelties: 1) a comprehensive environmental analysis based on Life cycle assessment (LCA) methodology aiming at evaluating the environmental impact of any LAES plant's component; 2) the evaluation of the intrinsic flexibility of multi-energy LAES in analogy with the reliability and robustness of a commercially available energy storage solution (Li-ion battery) by adopting a life cycle approach, assessing thus different case studies and scenarios. By overcoming the limitations presented in literature, the present work aims to demonstrate how: 1) the thermal energy storage systems must be properly accounted for when evaluating the environmental performance of the LAES and more in general of any thermo-mechanical energy storage systems; 2) the multi-energy capability of LAES can further enhance the environmental performance of the plant.

Thoroughly, this study focuses on the evaluation of the environmental impact of EESs by means of the LCA approach. The LCA metric was selected as it represents the most widely recognised methodology to evaluate environmental impact of product systems and services [40]. As mentioned above, a comparison between one established energy storage technology and a relatively novel technology was carried out. Among different and commercially available battery types, Li-ion battery is the leading option in terms of energy density, lifetime expectancy and the use of less environmentally intensive materials [41]; in addition to this, Li-ion battery withstand higher depth of discharge and can reach significantly high roundtrip efficiency [[42], [43], [44]]. The novel technology considered in this paper is represented by LAES. From a technological point of view, it can be considered a robust and well-established technical option, and some pilot plants are already operating [13,45]. However, its full potential has not been explored yet as LAES main advantage is to simultaneously provide electricity and cooling power from the same energy medium, namely liquid air, whereas other technologies require additional machines (e.g., chillers) to supply cooling power. LCA metrics for Li-ion battery and the LAES were calculated as part of the current work.

The paper structure is organized as follows. Section 2 presents and systematically describes the LCA methodology along with the different storage technologies investigated in the work. In Section 3 the results obtained from the LCA are presented and comparatively assessed; in Section 4 the main conclusions are drawn.

## 2 Materials and methods

### 2.1 System description

This section provides an overview of the electrical energy storage systems modelled in the LCA investigation: liquid air energy storage system and Li-ion battery.

#### 2.1.1 Liquid air energy storage (LAES)

**Fig. 2:**
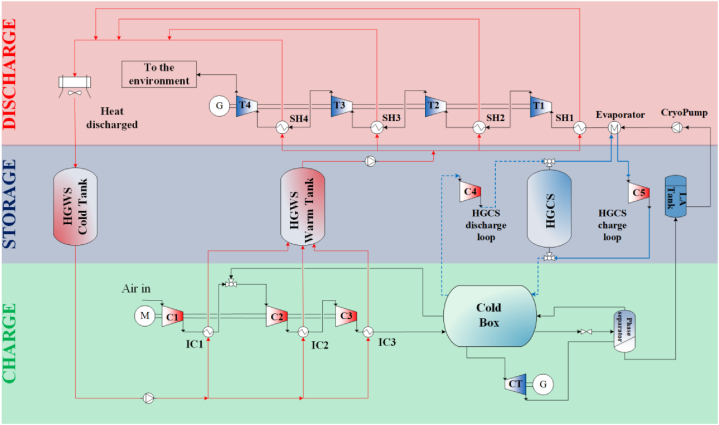
LAES process flow diagram process. Adapted from Ref. [23].

Fig. 2 illustrates the process flow diagram of the LAES system utilized for the LCA analysis. The system comprises five main sections: an air liquefaction cycle, a liquid air storage tank, high grade warm and cold storages, and a power recovery cycle. During the charge phase, surplus electricity produced from renewables is utilized to compress the working fluid in the liquefaction process, designed as a Kapitza thermodynamic cycle [11]. The waste heat generated during the air compression process is stored in a dedicated hot thermal energy storage system (high grade warm storage - HGWS) to be later utilized during the discharge phase. To this end, a sensible heat double tank configuration is employed using Therminol 66 as the heat transfer and storage medium. The working fluid undergoes the cooling process in the cold box and then it is throttled through an isenthalpic Joule-Thomson valve. Subsequently, the liquid phase is extracted in the phase separator and stored in a designed pressurized tank. During this period, the waste cold released by the liquid air regasification process and stored in a dedicated cold thermal energy storage is used for assisting the liquefaction of the ambient air in the cold box before it enters the isenthalpic expansion through the Joule-Thomson valve.

During the discharge phase, the liquid air of the power recovery unit is firstly pumped to the evaporator and then to the superheaters where the waste heat stored in the HGSW system increases the air temperature until reaching the designed turbine inlet temperature. Subsequently, the working fluid undergoes a four stages expansion coupled to an electric generator that feeds the electricity back to the grid. A cold thermal energy storage system (high grade cold storage - HGCS), namely a sensible heat based packed bed, is implemented to store the waste cold stream available at the air evaporation process, using nitrogen as heat transfer fluid. More technical information regarding this component can be found in Refs. [46,47].

The LAES is modelled after a 50 MW_e_/150 MWh_e_ grid-scale plant, with a power to energy ratio similar to that proposed in Ref. [48]. The LAES architecture depicted in Fig. 2 clearly illustrates in detail the different phases and components of the cryogenic thermo-mechanical energy storage system.

#### 2.1.2 Li-ion battery

Commercialized for the first time by Sony in 1990 [49], Li-ion batteries operate by utilizing Li-intercalation compounds, where lithium ions migrate through the electrolyte situated between two host structures functioning as the positive and negative electrodes [50]. The initial generation of Li-ion batteries enabled the storage of over double the energy capacity when compared to nickel or lead batteries of equivalent size and weight [51]. Over the years, improvement on materials have led to a consistent growth in terms of energy density (up to 200 Wh/kg), life cycles (as high as 10,000 cycles) and efficiency (which has reached values close to 95 %) [49]. The two key factors used to assess EESs are energy density and round trip efficiency because of the protracted operation time as well as volume and space constraints. For these reasons, Li-ion batteries represent one of the most promising battery types among the different electrochemical storage systems. Despite a widespread predominance of Li-ion batteries in the portable device market, where they account for more than 50 % of the total share, there are some challenges for making Li-ion batteries suitable to large scale applications. The main obstacle is represented by the high production cost (∼$900–1300 per kWh [51]) due to special packaging and internal overcharge protection circuits. However, looking from a storage point of view, Li-ion batteries are suitable for electric vehicle (EV) solutions thanks to the low self-discharge ratio (0.1–0.3 % per day) [51]. Furthermore, from an embodied energy perspective, due to a predominance of the use phase as compared with other life stages, Li-ion batteries are considered as environmental benign [42].

### 2.2 Life cycle assessment methodology (LCA)

**Fig. 3:**
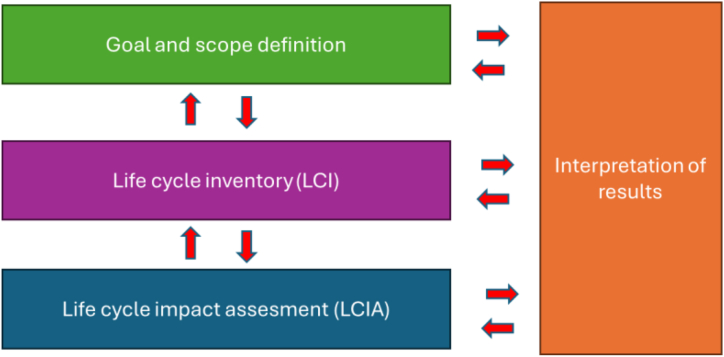
LCA methodology steps. Adapted from Ref. [52].

Life Cycle Assessment is a step-by-step methodology used to evaluate the environmental impact during the life cycle or a product/service. According to the UNE-EN ISO 14040:2006 and ISO 14044 standards, LCA involves four main stages: goal and scope definition, life cycle inventory, life cycle impact assessment and interpretation of results [52,53] as shown in Fig. 3. Each stage will be examined in detail in the following paragraphs.

#### 2.2.1 Goal and scope definition

**Fig. 4:**
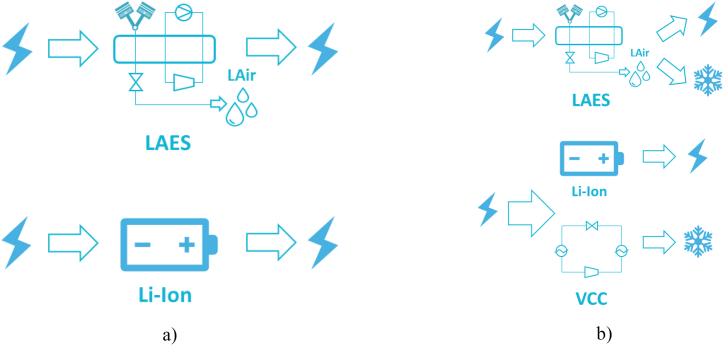
Conceptualization of the case studies under investigation – a) Full electric and b) cogeneration assets.

The objective of the study is to comparatively assess the environmental impact of two different energy storage technologies: Li-ion battery and LAES. As shown in Fig. 4, the utilization of the battery analogy constitutes the chosen approach for conducting a comprehensive comparative assessment among the previously delineated technologies. The establishment of an unbiased comparative framework holds intrinsic significance. As earlier indicated, the inherent potential of LAES to exhibit a dual output—comprising both electricity and cooling—necessitates a comprehensive dissection of the three phases, namely charging, storing, and discharging, explicated individually for every distinct energy storage technology. The layout of LAES is here simplified since the main aim is to display the key components and processes without further details.

As previously mentioned, LAES is capable to operate as multi-energy system, simultaneously delivering both electricity and cooling, which respect to the abovementioned function is considered as a co-product. Therefore, two different case studies were considered in the LCA analysis, as shown in Table 1. In the first case study, the LAES in full electric configuration, delivering only electricity as output, was comparatively assessed with a Li-ion battery of the same storage capacity of 150 MWh. On the other hand, when LAES is designed as a multi-energy system with the simultaneous delivery of electricity and cooling (case study 2), a system including a water-cooled vapour compression chiller (VCC) coupled with a Li-ion battery with the same storage capacity of the LAES (150 MWh) was introduced to have a fair comparison of two systems delivering the same energy outputs. Details on the sizing of the systems are described in Section 2.2.4.2 and reported in Table 3.

**Table 1:** Case studies for LAES and Li-ion battery.

Case study	LAES	Li-ion battery
# 1	This configuration aims to store and deliver only electricity. The round trip efficiency of the LAES is maximized using the HGCS to decrease the specific consumption of the liquefaction process (LAES charging).	The only discharge output is the electric energy. Li-ion battery is considered as the unique existing system.
# 2	This configuration aims at simultaneously delivering both electricity and cooling. A residential user that needs a cold temperature source of 7 °C is considered the main cooling load to be fulfilled for this case study. The cooling load is provided by two sources. The first one is the cooling available at the high grade cold storage recycling the waste cold from air regasification. Approximately 25 % of the cooling potential available at the high grade cold storage is provided to the residential's cooling load while the remaining 75 % is exploited by the air liquefier. The second source of cooling energy is represented by the air at the outlet of any expander stage, presenting an ideal temperature for district cooling of ∼0 °C. The cooling/electric power ratio is 0.39.	This configuration aims at producing both electricity and cooling energy. A water-cooled vapour compression chiller (VCC) is integrated with the Li-ion system to deliver the cooling energy required by the residential user.

**Table 2:** Share of the total weight for a typical Li-ion battery system [34].

Component	Share of the total weight [%]
Battery cell	60
Packaging	32
Battery management system (BMS)	4
Cooling system	4

**Table 3:** Use phase system parameters.

	Case study 1		Case study 2		
Technologies	Li-ion battery	LAES	Li-ion battery	LAES	Chiller
Power [MW_e_]	50	50	50	50	–
Cooling [MW_c_]	–	–	–	13	13
Capacity [MWh_e_]	150	150	150	150	–
Waste heat recovered [MW_th_]	–	30	–	45	–
Round trip efficiency [%]	66	60	66	43	–
COP [−]	–	–	–	–	4
Electricity input [MWh_e_]	2250000	2386309	2250000	3459925	143911
Cooling output [MWh_c_]	–	–	–	575645	575645

#### 2.2.2 Functional unit

This work assumes that both the energy storage technologies are charged during off-peak periods and discharged during peak load demand. Therefore, all the EESs are assumed to be charged by the same energy source and connected to the grid delivering a fixed electric power (i.e., 50 MW_e_) for a finite amount of time.

The functional unit is then defined as the delivery of 1 MWh of electricity output to the electrical grid. Such function should be accomplished over a lifetime of 30 years, meaning that the EESs must ensure the designated functionality throughout the whole lifetime. This may entail the necessity for replacement and maintenance of specific components or parts to uphold prolonged operational efficacy. While LAES performance was computed by a dedicated code developed by the authors in Matlab, data regarding Li-ion battery performance were retrieved from the literature.

#### 2.2.3 System boundaries definition

The present study assumes an approach “from cradle to grave”, namely raw material extraction, plant manufacturing and the use phase are included in the analysis. Therefore, the system boundaries include material inputs into the manufacturing and incorporate the use-phase energy and material inputs, namely the impacts for electric production mainly due to the charging of the EES systems, and the end-of-life processing and products. The plant decommissioning is left outside the system boundaries since for LAES there is lack of real data related to their end-of-life. Nevertheless, the disposal phase was considered for each component. In the case of LAES, steel components were considered to be disposed on inert land fill. Regarding the Li-ion battery cell, market values from Ecoinvent database were used which mainly considers pyrometallurgical and hydrometallurgical treatment for the disposal.

#### 2.2.4 Life cycle inventory (LCI)

In this stage, all the life cycle data included in the goal and scope were collected using different sources including literature, manufacturers data sheet, and simulation tools. Whereas life cycle data related to the Li-ion battery were mainly collected from existing available literature, LAES inventory data come from an internally developed numerical model previously deployed in many literature works (Refs. [23,54]).

##### 2.2.4.1 Manufacturing and disposal

The manufacturing and disposal stage data of the LAES were retrieved from catalogue of different components and existing literature works [39,55]. When not directly available, the weights of components were scaled according to the available data.

The inventory is a list of all substances involved in the LCA process. Each system was evaluated separately. Table 5 and Table 6 in the appendix section list the materials included in the LAES inventory for the full electric and the cogenerative configurations, respectively. The heat transfer fluid of the high grade warm storage is assumed to be a mixture of 73 % diphenylether and 27 % phenol [56]. The components nomenclature follows the one established in the process flow diagram in Fig. 2.

The total weight of the Li-ion battery was calculated considering an energy density of 140 Wh_e_/kg (Ref. [57]) whereas the single components’ weights were computed using the estimation reported by Carvalho et al. [34], as shown in Table 2.

The materials used to manufacture each component of the Li-Ion battery (Table 7 in the appendix section) and the vapour compression chiller serving a district cooling district (Table 8 in the appendix section) were retrieved and adapted from the Ecoinvent database [58] and from the studies published by Ellingsen et al. [59] and Gu et al. [60], respectively. In the latter case the quantity of material used for the manufacturing was scaled to correspond to the size considered in this study, namely a vapour compression chiller designed to have the same cooling output as the LAES in the cogeneration case study.

##### 2.2.4.2 Use phase

This study considers the impact of the different energy inputs during the “use phase”, namely the operation of the EES systems. Indeed, the effect of the energy inputs during the charging phase on the environmental performance might be a determinant factor, especially for EES characterised by low round trip efficiency values. The energy input of the Li-ion battery was calculated starting from the electrical output (i.e. 50 MW) and considering a conservative life-cycle energy efficiency of 66 %, assuming inverter, thermal management and cell efficiency losses [61]. The electric consumption of the chiller in the cogenerative configuration was estimated considering an average coefficient of performance (COP) of 4 [62]. Table 3 shows a summary of the electric consumption calculated for the different systems in the different case studies analysed.

#### 2.2.5 Life cycle impact assessment (LCIA)

The impact assessment was based on the Ecoinvent 3.8 database using the model “allocation at the point of substitution”. In this study, the main performance indicators used are the ReCiPe and the Global Warming potential (GWP). The ReCiPe indicator is based on an updated version of CML and Eco-indicator99 [63]. These indicators are based midpoint indicators which are converted though damaged pathways into endpoint indicators which includes: effect on human health, biodiversity, and resource scarcity. On the other hand, the impact expressed though GWP, express the ratio of the radiative damage force of greenhouse gases measured in kgCO2-eq. The effect of greenhouse gases can be evaluated as a long-term effect (GWP 100a) or short-term effect (GWP 20a) to the atmosphere. In this study the indicator GPW 100a was used to comparatively assess the results.

To the best of authors’ knowledge, the effect of the different energy mix for electricity generation and its carbon emissions implications were rarely considered in any studies regarding EES or in particular LAES. To this end, a sensitivity analysis regarding the effect of different sources of energy during the use phase was carried out considering different scenarios, as shown in Table 4.

**Table 4:** Case scenarios for LCA of LAES and Li-Ion battery.

Scenarios	Energy mix
1	Singapore
2	Spain
3	Brazil
4	France
5	100 % Hydropower
6	100 % Photovoltaic
7	100 % Wind
8	100 % Nuclear energy
9	RES energy mix (50 % wind, 30 % PV, 20 % hydro)
10	Nuclear and RES energy mix (50 % nuclear, 25 % wind, 15 % PV, 10 % hydro)

**Fig. 5:**
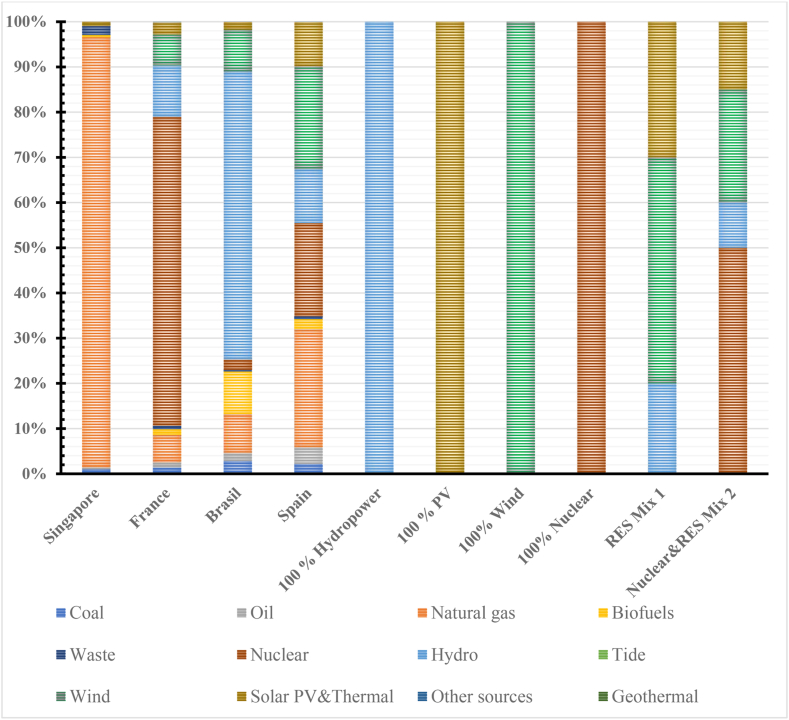
Electricity generation by primary energy source for the case scenarios [64].

The first four scenarios consider realistic case studies from national energy mixes. The energy mix is the one included in the Ecoinvent databased which is based on IEA electricity information 2014 [64]. The countries considered were selected to represent different cases and share of primary energy sources. The intermediate scenarios consider that the electricity is 100 % generated by either single renewable energy sources or nuclear energy. The last two scenarios consider hypothetical future energy sources for electricity generation. The first scenario is an ideal scenario including only a mix renewable energy sources whereas the second one presents a more realistic mix of nuclear energy (50 %) and renewable energy sources. A summary of the energy mixes for the different scenarios is shown in Fig. 5.

## 3 Results and discussion

This section illustrates the environmental impact of the LAES plant and Li-ion battery calculated according to the Life Cycle Impact Assessment (LCIA) method.

### 3.1 Case study 1 – full electric configuration

**Fig. 6:**
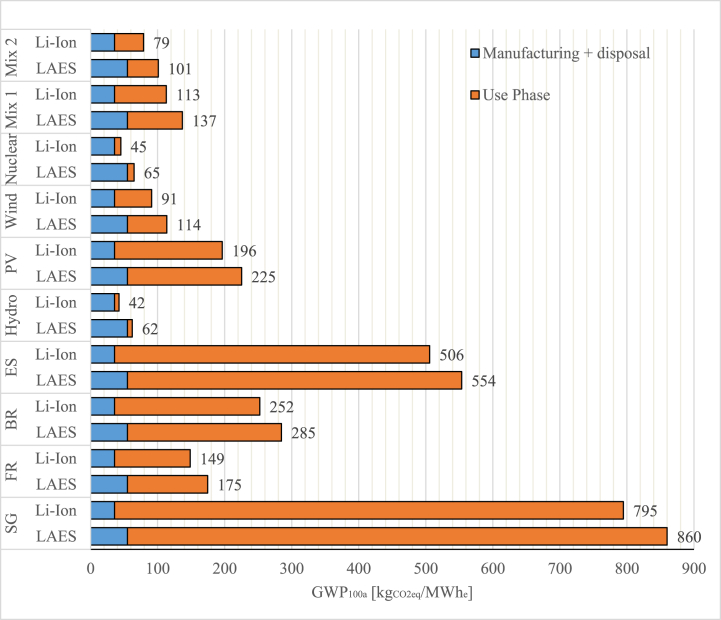
Case study 1 - GWP 100a impact category for the different energy mix considered.

Fig. 6 shows the results of the impact assessment of the two technologies using the indicator GWP 100a [kg_CO2eq_/MWh_e_]. Indeed, the main objective of this figure was to study the difference between the two systems under investigation in terms of their associated greenhouse gases long-term environmental effects. The results were allocated into separate categories for the manufacture and the use phases. Since in this case study the analysis was carried out for solely electricity generation as output, LAES was comparatively assessed in respect to a Li-ion battery.

The results show that in all the scenarios considered Li-ion battery has a lower impact during the whole life cycle. It is worth noting that especially in those scenarios heavily relying on fossil fuels as primary energy sources (e.g. Singapore and Spain energy mixes), the use phase shows the highest impact in the life cycle of the two technologies considered. For instance, considering Spanish energy mix, Li-ion battery and LAES produce a GWP of 506 and 554 kgCO_2eq_/MWh_e_, respectively whereas in Singapore the GWP impact reaches 795 and 860 kgCO_2eq_/MWh_e_ for the Li-ion battery and LAES, respectively. In a 100 % renewables scenario perspective, the impact of the use phase becomes less significant, being the manufacturing and the disposal the main sources of environmental impact during the whole life-cycle. The lowest impact considers a scenario with 100 % energy supplied by nuclear energy sources where the impact decreases to 45 and 65 kgCO_2eq_/MWh_e_ for the Li-ion battery and LAES, respectively. Notably, for GWP indicator, the lower round trip efficiency of LAES (∼60 %) is a less significant factor if hydro or wind energy is assumed to be the only input for the liquefaction of air (charging phase).

**Fig. 7:**
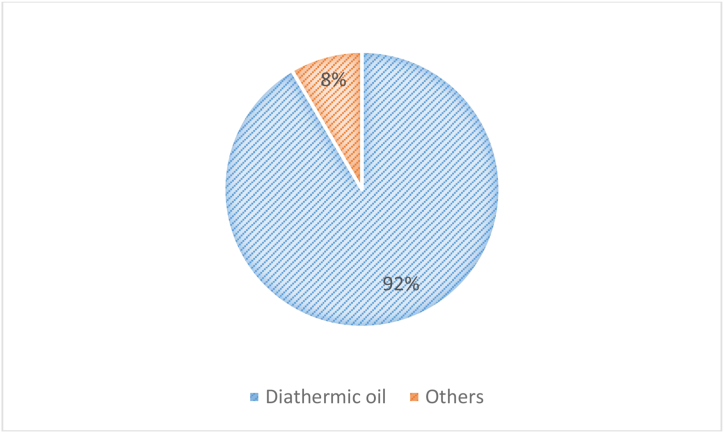
Case study 1 - Share of the main “Manufacturing&Disposal” components.

Interestingly, Fig. 7 shows that, analysing the “fossil fuel free” scenarios, the diathermic oil, used to recover the heat of compression during the charging phase to increase the turbine inlet temperature at the discharge phase, has the highest share of the environmental impact for the LAES “Manufacturing&Disposal” phase. Thermodynamically, to harness a total of 30 MW_th_ of thermal power recovered from air compression, the LAES requires to store approximately 3000 tons of diathermic oil. This outcome suggests that one of the main hotspots to further improve the environmental impact is the potential replacement of the current technology employed for the high-grade warm storage. As a promising alternative solution, already technically proposed by some works in literature (Refs [65,66]), different storage materials including molten salts, or air might trigger a significant enhancement of the sustainability performance of LAES technology.

**Fig. 8:**
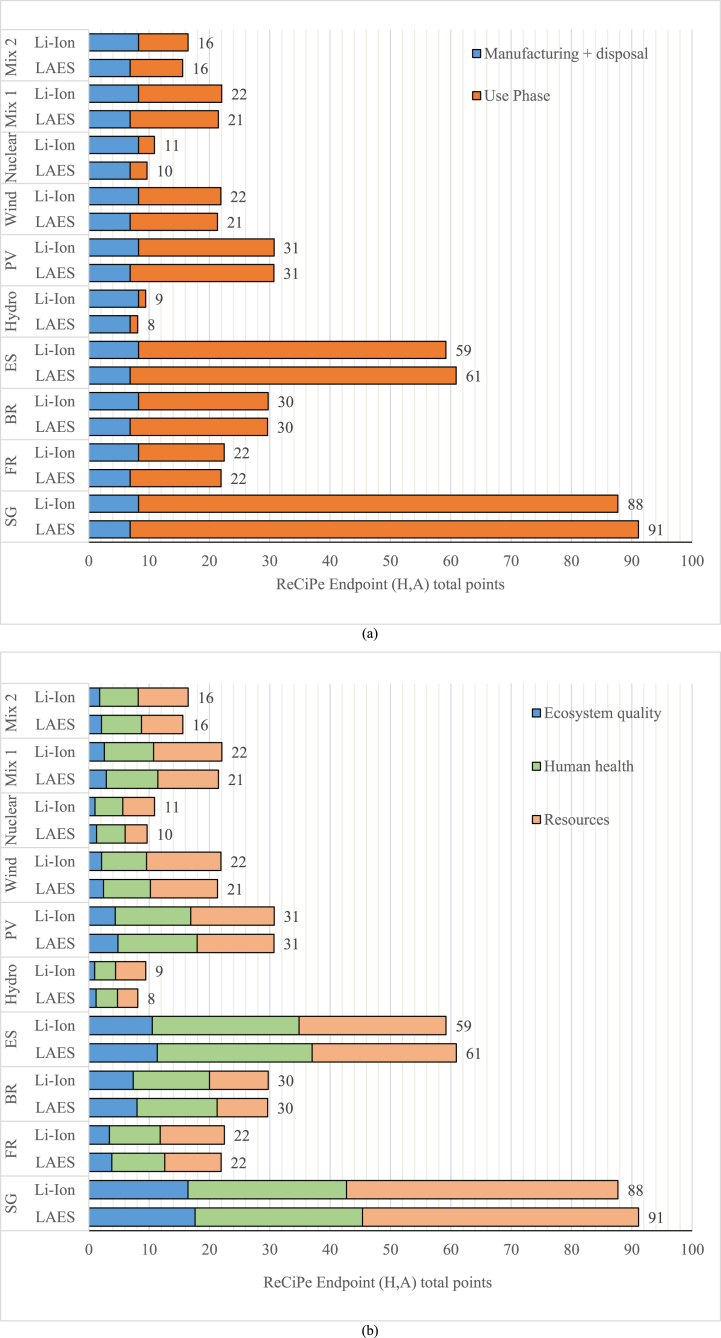
Scenario 1 – ReCiPe indicators for the different energy mix considered.

Furthermore, the impact of the manufacturing and use phases was assessed using the ReCiPe indicators (Fig. 8a) for all the scenarios considered. In this case Li-ion battery and LAES have a similar environmental impact. The highest impact is observed when considering the Singapore energy mix scenario, where Li-ion battery and LAES account for 88 and 91 points, respectively. However, confirming the former results for GWP indicator, the operational stage has the highest impact on the total life cycle in most of the scenarios, especially the ones with a high share of fossil fuel in the energy mix. The operational stage has minimal impact in scenarios where the two systems harness nuclear or hydropower sources. Notably, LAES shows slightly lower impact points in scenarios with a high share of renewable energy sources. For instance, in the hydropower scenario, LAES accounts for 8 impact points, compared to 9 for the Li-ion battery. Fig. 8b shows that for both technologies the share of impact on different categories is similar with the highest impacts on human health and resources. The primary difference between LAES and Li-ion battery lies in their impact on ecosystem quality, with LAES having a slightly higher impact across the entire life cycle, including the operational stage.

### 3.2 Case study 2 – Electricity and cooling cogeneration

**Fig. 9:**
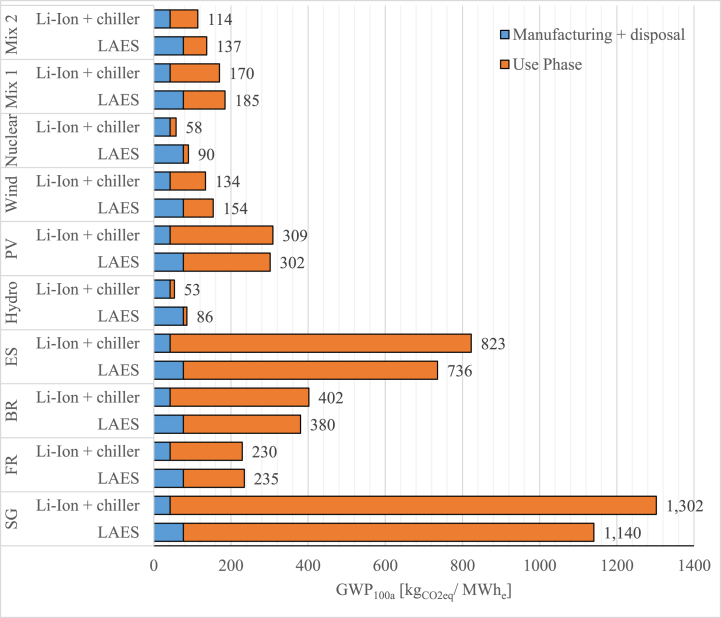
Case study 2 - GWP 100a impact category for the different energy mix considered.

In this case study the EES systems examined are assumed to be operating within a cogeneration asset framework. Through its discharge phase and the high grade cold storage system, LAES system supplies electricity to the grid while also providing cooling energy to a district cooling application, contributing thus to the overall energy system resilience and sustainability. Likewise, since the standalone Li-ion battery does not exhibit this cogeneration capability, it was integrated with a vapour compression chiller to provide both electricity and cooling. Fig. 9 shows the results of the impact assessment carried out for the cogenerative configuration using the GWP 100a indicator.

Compared to the full electric case study (Fig. 6), it can be noted that the manufacturing and disposal phases of LAES shows a higher environmental impact. This is due to a significant decrease of the LAES’ round trip efficiency (43 % vs 60 % in full electric case study), which necessitates larger component sizing to deliver the same electric power output. Indeed, the supply of the cooling energy is coming at stake of the global efficiency since part of the energy stored is conveyed to produce cooling instead of being recycled to support the air liquefaction process. Nevertheless, cogenerative LAES shows a lower environmental impact when the first four scenarios (energy mixes of national countries) are considered. This outcome reflects the fact that LAES shows a lower electricity consumption to provide both electricity and cooling services. Such a capability has a clear significant benefit especially when the GWP impact of electricity generation is high, i.e. for scenarios with high share of fossil fuels. For instance, considering Singapore's energy mix, the environmental impact of the Li-ion battery and chiller decreases from 1302 kg_CO2eq_/MWh_e_ to 1140 kg_CO2eq_/MWh_e_ with a cogenerative LAES.

Conversely, in a 100 % renewable scenario or in an energy mix characterized by a high share of renewable (mix 1 or mix 2), Li-ion battery and chiller show a lower impact since the use phase become less significant as compared to the global life cycle impact. For instance, considering the 100 % hydropower scenario, Li-ion battery and chiller achieve the lowest impact of 58 kg CO_2eq_/MWh_e_. However, as for the full electric case study, the environmental impact of LAES could be reduced introducing a more sustainable solution for the high-grade warm storage.

**Fig. 10:**
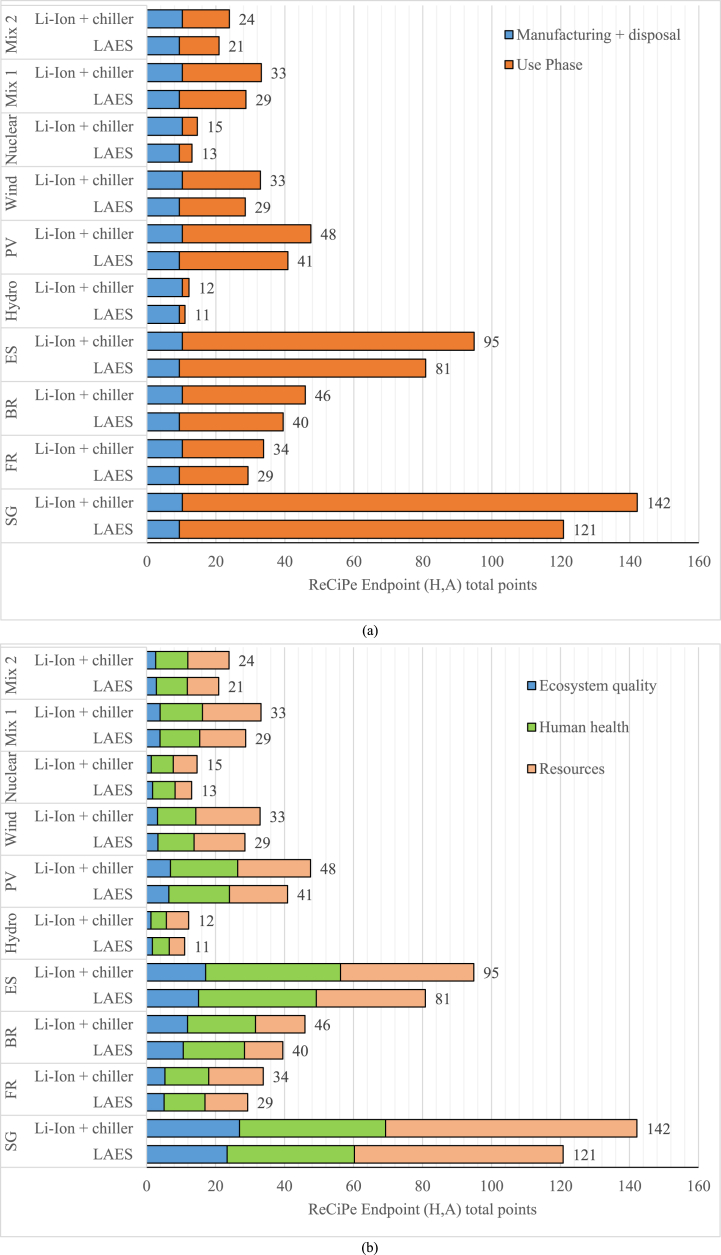
Scenario 2 – ReCiPe indicators for the different energy mix considered.

Fig. 10 shows the environmental impact according to the ReCiPe indicators. Highlighting the importance of comparative LCA using different indicators, the results show that LAES has lower environmental impact points compared to the Li-ion battery in all the scenarios considered. Indeed, as shown in Fig. 10a, since the impact points for the manufacturing and disposal have a similar value across both EES solutions, the lower energy consumption during the use phase of the cogenerative LAES, which provides both electricity and cooling, leads to a reduced overall environmental impact. Furthermore, as in the full electric case study, the gap between the two systems is more significant in the scenarios where the energy mix heavily relies on fossil fuels (Singapore and Spain). Considering the energy mix of Singapore, LAES environmentally outperforms the Li-ion battery and chiller configuration, with 121 total points compared to 142. In a 100 % renewable scenario or scenarios with a high share of renewables, the gap become less relevant due to the lower influence of the use phase in the total impact. Fig. 10b shows that the higher impact points and hazards are due to the human health and exploitation of resources indicators, presenting lower values for the LAES system. The findings from the cogenerative case study reveal that employing LAES in this setup holds significant promise as an EES technology capable of supplying both electricity and cooling, despite experiencing a reduction in round trip efficiency. Notably, the environmental impacts of storing electricity and subsequently converting it into cooling overcome those of utilizing a cogeneration system. These results indicate that the cold energy generated and released in the LAES possesses considerable value for direct application in cooling processes. In a prospective scenario, marked by escalating demands for both cooling and electricity and coupled with constraints on lithium resources, LAES emerges as a viable option with clear environmental advantages.

## 4 Conclusions

The current study comparatively evaluates the environmental performance of LAES and Li-ion battery by means of LCA under different technical configurations and energy mix scenarios. At first, the comparison highlights the massive role played by the use phase, especially in those scenarios characterized by high share of fossil fuel in the energy mix. In these scenarios such plants are characterised by a significantly higher impact for the use phase, with an average share higher than 90 %, which annihilate impacts related to the manufacturing and disposal phase of the plant itself. Secondly, to some extent, round trip efficiency is not the only driven parameter since the study demonstrates that a well-balanced production of electricity and cooling power can lead to different outcomes and interpretations. Furthermore, the analysis demonstrates that despite being still a relatively new technology, LAES has proved to be environmentally competitive especially when operated in cogeneration asset. A key role is played by the production of cooling energy, which is capable to mirror the impact related to the electricity consumption in full electric configuration, leading to significant advantage compared to Li-ion battery where cooling power cannot be considered as co-product. Indeed, analysing the full electric case study, Li-ion battery environmentally outperforms LAES in all the scenarios considered with the lowest impact showed in a 100 % renewable scenario (45 vs 65 kgCO_2eq_/MWh_e_, respectively). Conversely, despite its lower round trip efficiency, the multi-energy LAES presents a lower environmental impact, particularly in fossil fuel-dominated scenarios (e.g., Singapore), compared to the integrated Li-ion battery and chiller systems (1140 kg_CO2eq_/MWh_e_ vs 1302 kg_CO2eq_/MWh_e_).

The LCA analysis also shows that especially for the scenarios characterized by high share of renewable in the energy mix, the highest share of the LAES environmental impact in terms of GWP is due to the utilization of the diathermic oil as a heat transfer fluid and storage medium for the thermal energy storage collecting the heat of compression (HGWS). This impact might be mitigated by the introduction of novel configurations based on a different storage medium for the high grade warm storage (i.e. pressurized water and molten salt). Furthermore, the potential to further exploit the unused waste heat available in the HGWS could lead to further economic and environmental benefits that a future study might systematically capture and numerically evaluate.

As a final remark, such comparison would need more investigations in terms of available LCA study as well as data from the field. The best-case scenario would be to develop a detailed LCA analysis for the two different technologies in order to be aligned at every step.

## CRediT authorship contribution statement

**Alessio Tafone:** Writing – review & editing, Writing – original draft, Methodology, Investigation, Formal analysis, Data curation, Conceptualization. **Emiliano Borri:** Writing – original draft, Visualization, Software, Investigation, Formal analysis. **Luisa F. Cabeza:** Writing – review & editing, Supervision. **Alessandro Romagnoli:** Writing – review & editing, Supervision.

## Declaration of competing interest

The authors declare that they have no known competing financial interests or personal relationships that could have appeared to influence the work reported in this paper.

## Figures and Tables

**Table 5:** Inventory list for the LAES system in Case study 1.

Module	Components	Sub-Components	Materials from Ecoinvent	Quantity [kg]	Expected lifetime (years)
Charging unit	Compressor C1		Steel, low-alloyed	77502	30
	Compressor C2			77502	30
	Compressor C3			77502	30
	Aftercooler AFC1			1240	30
	Intercooler IC			1297	30
	Aftercooler AFC2			1416	30
	Cold Box		Aluminium alloy, AlMg3	36294	30
	Cryoturbine CT		Steel, low-alloyed	4332	30
	Phase Separator			3950	30
Liquid air tank	Liquid air tank	Inner/Outer vessel	Reinforcing steel	535560	30
		Insulation	Expanded perlite	82824	30
Thermal energy storage	HGWS	Warm Tank - Vessel	Steel, low-alloyed	570465	30
		Cold Tank - Vessel		504150	30
	HGCS	Vessel		754019	30
		Insulation	Expanded perlite	91926.378	30
		Storage material	Gravel, round	3359000	30
	Thermal oil		Diphenylether-compound/phenol	3000000	15
Discharge	Cryopump		Steel, low-alloyed	17375	30
	Evaporator			504238	30
	Superheater SH1			2132	30
	Superheater SH2			1644	30
	Superheater SH3			1600	30
	Superheater SH4			1585	30
	Power Turbine T1			24975	30
	Power Turbine T2			24641	30
	Power Turbine T3			24586	30
	Power Turbine T4			24575	30

**Table 6:** Inventory list for the LAES system in Case study 2.

Module	Components	Sub-Components	Materials from Ecoinvent	Quantity [kg]	Expected lifetime (years)
Charge phase	Compressor C1		**Materials from Ecoinvent**	117171	30
	Compressor C2		Steel, low-alloyed	117171	30
	Compressor C3		Steel, low-alloyed	117171	30
	Aftercooler AFC1		Steel, low-alloyed	1446	30
	Intercooler IC		Steel, low-alloyed	1821	30
	Aftercooler AFC2		Steel, low-alloyed	1953	30
	Cold Box		Aluminium alloy, AlMg3	53569	30
	Cryoturbine CT		Aluminium alloy, AlMg3	10882	30
	Phase Separator		Steel, low-alloyed	6419	30
Liquid air tank	Liquid air tank	Inner/Outer vessel	Reinforcing steel	679558	30
		Insulation	Reinforcing steel	104100	30
Thermal energy storage	HGWS	Warm Tank - Vessel	Expanded perlite	881284	30
		Cold Tank - Vessel	Steel, low-alloyed	793168	30
	HGCS	Vessel	carbon steel/low temperature resistant austenitic steel	754019	30
		Insulation	Expanded perlite	115491	30
		Storage material	Expanded perlite	3359000	30
	Thermal oil		Gravel, round	4645000	15
Discharge phase	Cryopump		Diphenylether-compound/phenol	21917	30
	Evaporator			634246	30
	Superheater SH1			1588	30
	Superheater SH2			1712	30
	Superheater SH3			1636	30
	Superheater SH4			1610	30
	Power Turbine T1			586	30
	Power Turbine T2			518	30
	Power Turbine T3			490	30
	Power Turbine T4			481	30

**Table 7:** Inventory list for the Li-ion battery system.

Module	Components	Sub-Components	Materials from Ecoinvent	Quantity [kg]	Expected lifetime (years)
Cell			battery cell, Li-ion	1071428	10
Packaging			battery module packaging	571428	20
BMS			battery management system	71428	20
Cooling system	radiator	–	aluminium alloy, AlMg3	62142	20
	mainifolds	–	aluminium alloy, AlMg3	2714	20
	clamps	–	steel, low‐alloyed	1642	20
	pipe	Pipe fitting plastic	polyvinylchloride	51	20
		Pipe Fitting rubber	synthetic rubber	17	20
	thermal pad	Thermal pad, glass fibre	glass fibre	142	20
		Thermal pad, silicon	silicon, electronic grade	428	20
		Thermal pad, ABS	acrylonitrile‐butadiene‐styrene copolymer	857	20
	coolant		ethylene glycol	3428	20

**Table 8:** Inventory list for the vapour compression chiller in Scenario 2.

Module	Ecoinvent Materials	Quantity [kg]	Expected lifetime (years)
water-cooled chiller	steel, low-alloyed	68981	20
	copper, cathode	18395	20
	aluminium alloy, AlMg3	1230	20
	refrigerant R134a	3367	20
cooling tower	steel, low-alloyed	18915	15
	polyvinylchloride, bulk polymerised	18915	15
cooling water pump	steel, low-alloyed	13148	15
